# Systematic identification of the interventional mechanism of Qingfei Xiaoyan Wan (QFXYW) in treatment of the cytokine storm in acute lung injury using transcriptomics-based system pharmacological analyses

**DOI:** 10.1080/13880209.2022.2055090

**Published:** 2022-03-31

**Authors:** Jing-Yi Hou, Jia-Rong Wu, Yi-Bing Chen, Dong Xu, Shu Liu, Dan-dan Shang, Guan-Wei Fan, Yuan-Lu Cui

**Affiliations:** a State Key Laboratory of Component-based Chinese Medicine, Tianjin University of Traditional Chinese Medicine, Tianjin, People’s Republic of China; b Tianjin Key Laboratory of Transformation of Traditional Chinese Medicine, First Teaching Hospital of Tianjin University of Traditional Chinese Medicine, Tianjin, China; c Tianjin Zhongxin Pharmaceutical Group Corporation Limited Darentang Pharmaceutical Factory, Tianjin, China

**Keywords:** Anti-inflammatory, traditional Chinese medicine, protein array analysis, protein-protein interaction network

## Abstract

**Context:**

Acute lung injury (ALI) is a complex, severe inflammation disease with high mortality, and there is no specific and effective treatment for ALI. Qingfei Xiaoyan Wan (QFXYW) has been widely used to treat lung-related diseases for centuries.

**Objective:**

This study evaluates the potential effects and elucidates the therapeutic mechanism of QFXYW against LPS induced ALI in mice.

**Materials and methods:**

BALB/c Mice in each group were first orally administered medicines (0.9% saline solution for the control group, 0.5 mg/kg Dexamethasone, or 1.3, 2.6, 5.2 g/kg QFXYW), after 4 h, the groups were injected LPS (1.0 mg/kg) to induce ALI, then the same medicines were administered repeatedly. The transcriptomics-based system pharmacological analyses were applied to screen the hub genes, RT-PCR, ELISA, and protein array assay was applied to verify the predicted hub genes and key pathways.

**Results:**

QFXYW significantly decreased the number of leukocytes from (6.34 ± 0.51) × 10^5^/mL to (4.01 ± 0.11) × 10^5^/mL, accompanied by the neutrophil from (1.41 ± 0.19) × 10^5^/mL to (0.77 ± 0.10) × 10^5^/mL in bronchoalveolar lavage fluid (BALF). Based on Degree of node connection (Degree) and BottleNeck (BN), important parameters of network topology, the protein-protein interaction (PPI) network screened hub genes, including IL-6, TNF-α, CCL2, TLR2, CXCL1, and MMP-9. The results of RT-PCR, ELISA, and protein chip assay revealed that QFXYW could effectively inhibit ALI via multiple key targets and the cytokine-cytokine signalling pathway.

**Conclusions:**

This study showed that QFXYW decreased the number of leukocytes and neutrophils by attenuating inflammatory response, which provides an important basis for the use of QFXYW in the treatment of ALI.

## Introduction

Acute lung injury (ALI) is a comprehensive reflection of pulmonary inflammation caused by various pathogenic factors inside and outside the lung (Wang et al. 2020a), mainly characterised by infiltration and exudation of many inflammatory cells, acute inflammation of the alveolar epithelium, and alveolar and interstitial lung edema (Liu et al. 2020; Ma et al. 2021). The main clinical manifestations are acute progressive hypoxaemia respiratory distress, and non-cardiac pulmonary edema (Kumar 2020), which lead to respiratory failure with a fatality rate as high as 30–50% (Eves et al. 2013), posing a serious concern. Currently, ALI is mainly treated by reducing inflammation (Han et al. 2018), with anti-inflammatory drugs, such as corticosteroids (Nasralla and Abboud 2020), aspirin (Chen et al. 2020a), salbutamol (Cardoso-Sousa et al. 2019), and ketoconazole (Schilling et al. 2001). Although these drugs have certain therapeutic effects, adverse effects such as peptic ulcer, acute kidney injury, irregular heartbeat, and articular pain may hinder a satisfactory therapeutic effect (Fasolino et al. 2020; Liang et al. 2020).

Unlike the aforementioned chemical drugs, the traditional Chinese medicine (TCM) formulas are composed of a variety of Chinese Medicine Materials (CMMs) that have multi-component, multi-target, and multi-pathway characteristics (Li et al. 2018; Sheu et al. 2020). Qingfei Xiaoyan Wan (QFXYW), derived from “Maxing Shigan Decoction,” which was proposed by Zhang Zhongjing from the Han Dynasty and approved by the China Food and Drug Administration (NO. Z20103031), is mainly composed of eight components of TCM, namely, *Ephedra sinica* Stapf (Ephedraceae)*, Prunus armeniaca* L. (Rosaceae)*, Arctium lappa* L. (Asteraceae)*, Lepidium apetalum* Willd. (Brassicaceae), calculus bovis (Bovidae)*, Cornu Saiga tatarica Linnaeus* (Bovidae)*, Pheretima vulgaris* Chen (Megascolecidae), and *Gypsum Fibrosum* (Hou et al. 2016). This formula has been used for more than 30 years and has shown good therapeutic effects on cough, asthma, immune regulation, and antiviral activity in the clinic (Zhao et al. 2013; Hou et al. 2014). However, its therapeutic mechanism in the treatment of ALI has not been fully elucidated, necessitating further study and analysis.

Transcriptomics, also known as gene expression profiling, might explain the gene regulation mechanism of complex diseases, screen potential target genes and pathways through a variety of computational methods, and maybe successfully applied to reveal potential biomarkers or further explore the treatment of complex diseases (Kersch et al. 2020; Shockley et al. 2020). System pharmacology perfectly integrates the concepts and methods of system biology, bioinformatics, and pharmacology, focussing on a combination of multiple components, targets, and pathways to investigate the systematic mechanism of TCM (Zhao et al. 2018; Shu et al. 2019). The combination of transcriptomics and system pharmacology reveals the inheritable and genetic changes throughout the life process system, which is consistent with the principles of TCM that utilises multiple ingredients and targets, as a whole system in the treatment of diseases (Ding et al. 2020; Chen et al. 2020b).

In this study, we explored the therapeutic mechanism of QFXYW in the treatment of ALI via transcriptomics-based combined with system pharmacological analyses. After the development of ALI, transcriptomics analysis was applied to detect the genetic changes, and hub targets were screened out based on the system pharmacology analysis. Functional analysis was subsequently performed to predict the key pathways involved in the treatment of ALI with QFXYW. Finally, reverse transcription-quantitative polymerase chain reaction (RT-qPCR), enzyme-linked immunosorbent assay (ELISA), and protein array assay were used to verify the accuracy of the predicted results. This study provides a systematic investigation method to explain the biomarkers of QFXYW in the treatment of ALI, and attempts to elucidate the mechanism of QFXYW in the treatment of ALI.

## Materials and methods

### Materials

QFXYW was provided by Tianjin Zhongxin Pharmaceutical Group Co., Ltd. Darentang Pharmaceutical Factory (Tianjin, China, batch number: 9000102), and the ingredients were quantified using a UPLC-MS system as previously reported (Hou et al. 2016). Dexamethasone (DEX) was purchased from Bosen Bio-Pharmaceutical Co., Ltd. (Xi’an, China). Lipopolysacharide (LPS) was purchased from Sigma-Aldrich Co. (St. Louis, MO, USA). The interleukin (IL)-6 and tumour necrosis factor (TNF)-α Mouse ELISA kits were purchased from eBioscience (San Diego, CA, USA). The bicinchoninic acid (BCA) protein assay kit was purchased from Thermo Fisher Scientific (Waltham, MA, USA). Leukocyte dilution was supplied by Beijing Solarbio Technology Co., Ltd. (Beijing, China). The UNIQ-10 column Trizol total RNA extraction kit was obtained from Sangon Biological Engineering Technology & Services Co., Ltd (Shanghai, China). PrimeScript RT Master Mix and TB Green Premix Ex Taq II were purchased from TaKaRa Co. (Kyoto, Japan). The Mouse XL Cytokine Array kit was purchased from R&D Systems (ARY028, MN, USA).

### Animals and LPS-induced ALI

Male BALB/c mice were obtained from Beijing Sibeifu Experimental Animal Technology Co., Ltd. Certificate No. SCXK2016-0002, Beijing, China. All animal studies were performed in accordance with the guidelines of the National Institutes of Health (NIH), and the protocol was approved by the animal ethics committee of Tianjin University of Traditional Chinese Medicine (Labtcmdds-2018-N03). The results of the experiments are reported in accordance with the Animal Research: Reporting *in vivo* experiments (ARRIVE) guidelines and in agreement with the recommendations for good publishing practice in physiology (Persson 2019; Percie Du Sert et al. 2020). All animals were placed on adaptive feeding for 3 days to acclimatise them to the laboratory conditions. The mice were housed individually in cages and maintained under standard conditions of alternating 12 h light/dark cycles at a constant temperature of 24 ± 1 °C and relative humidity of 55 ± 5%. The 8–10-week-old (14–16 g), mice were randomly divided into the following six groups (*n* = 10, each group): control group, ALI group, ALI + DEX group, and three ALI + QFXYW groups (1.3, 2.6, and 5.2 g/kg). The mice in each group were first orally administered the respective medicines (0.5 mg/kg DEX or 1.3, 2.6, or 5.2 g/kg), and the control and ALI groups were administered an equivalent volume (0.2 mL/10 g) of 0.9% saline solution. After 4 h, saline solution was administered to the control group, and the remaining groups were administered LPS (1.0 mg/kg) to induce ALI, following which, the same medicines were repeated in each group. The animals in each group were subsequently administered isoflurane to collect blood from the retro-orbital sinus; the serum was separated and stored at −20 °C for subsequent experiments. The mice were then euthanized humanely through an overdose of anaesthesia, and the lung tissues were harvested.

### Histopathological evaluation of the lung tissue

To evaluate the histopathological changes in the lung tissue, the tissues were washed with cold phosphate buffered saline (PBS) solution three times and fixed in 4% paraformaldehyde, embedded in paraffin, cut into 4 μm sections, and stained with haematoxylin and eosin (H&E). Pathological changes in the lung tissues were examined using a light microscope.

### Bronchoalveolar lavage fluid (BALF) analysis

BALF was collected by gentle washing and perfusion of the lung tissue three times with cold PBS until the colour of lung tissue changed from red to white. The BALF liquid was collected and centrifuged (1000 rpm, 3 min, 4 °C) to filter the cells, and the supernatant was used for subsequent cytokine detection. The cells were then resuspended with leukocyte dilution and centrifuged to remove the impurities, and the precipitate was diluted with phenol red-free Dulbecco’s Modified Eagle’s medium (DMEM) to detect the total number of leukocytes and neutrophils.

### Transcriptomics analysis

#### RNA isolating and sequencing

The total RNA was extracted from the lung tissue and purified using the Trizol reagent kit following the manufacturer’s instructions (Huang et al. 2020). The concentration of RNA in each sample was quantified using the NanoPhotometer (Implen, Los Angeles, CA, USA). The integrity of the RNA was detected with the Agilent 2100 bioanalyzer (San Diego, CA, USA). A total of 1 μg of RNA per sample was used as input material for the RNA sample preparations. Novegene (Beijing, China) constructed the cDNA libraries and RT-qPCR was performed to accurately quantify the cDNA libraries. The cDNA sequencing libraries were generated on an Illumina HiSeq platform (Illumina Inc., San Diego, CA, USA) according to the manufacturer’s protocol.

#### Data analysis for gene expression

Differential expression analysis of two groups was performed using the DESeq2 in the R package (1.16.1) (Nagy and Győrffy 2021). DESeq2 provides statistical methods for confirming differential gene expression analysis based on the negative binomial distribution. The resulting *p*-values were adjusted using Benjamini and Hochberg’s approach for controlling the false discovery rate. Genes with an adjusted *p*-value < 0.05 according to the DESeq2 were assigned as differentially expressed.

#### Functional enrichment analysis

The DAVID, an online platform, was used to perform the pathway enrichment analysis to identify the Kyoto Encyclopaedia of Genes and Genomes (KEGG) and Gene Oncology (GO) pathways with differentially expressed genes (DEGs); terms with an adjusted *p*-value <0.05 were considered statistically significant.

#### Protein-protein interaction (PPI) construction and hub gene analysis

We first used the STRING online platform (https://string-db.org/) to construct the PPI network (combined score > 0.7) for the top 500 DEGs in the ALI vs. control groups, and 266 DEGs in the QFXYW vs. ALI groups, and imported it into the Cytoscape 3.2.1 platform for the Merge analysis. The plug-in CytoHubba (http://apps.cytoscape.org/cytohubba) was used to calculate the topological characteristic parameters of the nodes in the PPI network to screen out the hub genes (Zhu et al. 2020).

#### Reverse-transcription quantitative polymerase chain reaction (RT-qPCR)

The total RNA was isolated using the UNIQ-10 column Trizol total RNA extraction kit according to the manufacturers’ instructions. cDNA was generated using the High-Capacity cDNA PrimeScript™ Reverse Transcription Kit at 37 °C for 15 min, 85 °C for 5 sec, and 4 °C. PCR amplification was subsequently performed using TB Green Premix Ex Taq II. The primers used for this experiment were mouse β-actin, IL-6, TNF-α, CCL2, TLR2, IL-10, IL-1β, CXCL10, CXCL1, CXCL2, and MMP-9, as shown in Table 1. The experiments were conducted in triplicate for each sample, and the 2^-ΔΔCT^ method was used to calculate the relative expression of mRNA.

**Table 1.** Primers for RT-qPCR.

Gene	Sequence (5’-3’)	PCR product (bp)
β-actin (NM_007393.3)	Forward: TGTTACCAACTGGGACGACA	165
	Reverse: GGGGTGTTGAAGGTCTCAAA	
IL-6 (NM_031168.1)	Forward: TCCAGTTGCCTTCTTGGGAC	140
	Reverse: GTGTAATTAAGCCTCCGACTTG	
TNF-α (NM_013693.2)	Forward: TAGCCAGGAGGGAGAACAGA	127
	Reverse: TTTTCTGGAGGGAGATGTGG	
TLR2 (NM_011905.3)	Forward: AAGAGGAAGCCCAAGAAAGC	80
	Reverse: CAATGGGAATCCTGCTCACT	
CCL2	Forward: CCCAATGAGTAGGCTGGAGA	125
(NM_011333.3)	Reverse: TCTGGACCCATTCCTTCTTG	
CXCL1	Forward: TGTTGTGCGAAAAGAAGTGC	91
(NM_008176.3)	Reverse: TACAAACACAGCCTCCCACA	
CXCL2	Forward: AAGTTTGCCTTGACCCTGAA	180
(NM_009140.2)	Reverse: AGGCACATCAGGTACGATCC	
CXCL10	Forward: TCCTTGTCCTCCCTAGCTCA	124
(NM_021274.2)	Reverse: ATAACCCCTTGGGAAGATGG	
IL-1β	Forward: GACCTTCCAGGATGAGGACA	183
(NM_008361.3)	Reverse: AGCTCATATGGGTCCGACAG	
MMP-9	Forward: TGAATCAGCTGGCTTTTGTG	191
(NM_013599.4)	Reverse: ACCTTCCAGTAGGGGCAACT	

#### Measurement of cytokines levels

The collected serum, lung tissue homogenate, and BALF supernatant were stored at −80 °C until further use. The levels of IL-6 and TNF-α proteins in the serum, lung tissues, and BALF were quantified using the mouse ELISA kits according to the manufacturer’s instructions.

#### Protein array analysis

The differences in the expression of 111 inflammatory proteins were analysed in the serum and lung tissues, according to the instructions of the manufacture of the Proteome Profiler Array (R&D Systems; Minneapolis, MN, USA), a mouse antibody protein array kit. Briefly, the protein array membrane was first sealed in a blocking solution for 1 h and then incubated overnight with the serum or tissue protein at 4 °C. After repeated washing, the samples were incubated with an antibody for 1 h and then reacted with HRP-streptavidin for 30 min. Next, the protein array membrane was reacted with the chemical reagent for 1 min in darkness, and exposed to an X-ray film to obtain a protein array.

#### Statistical analysis

All data were analysed using Origin Pro 8.0 software and are expressed as mean ± standard error (Mean ± SE). Mean values of different groups were compared using one-way ANOVA. *p*-values < 0.05 were considered statistically significant.

## Results

### QFXYW exerts protective effects against LPS-induced ALI *in vivo*

**Figure 1.**
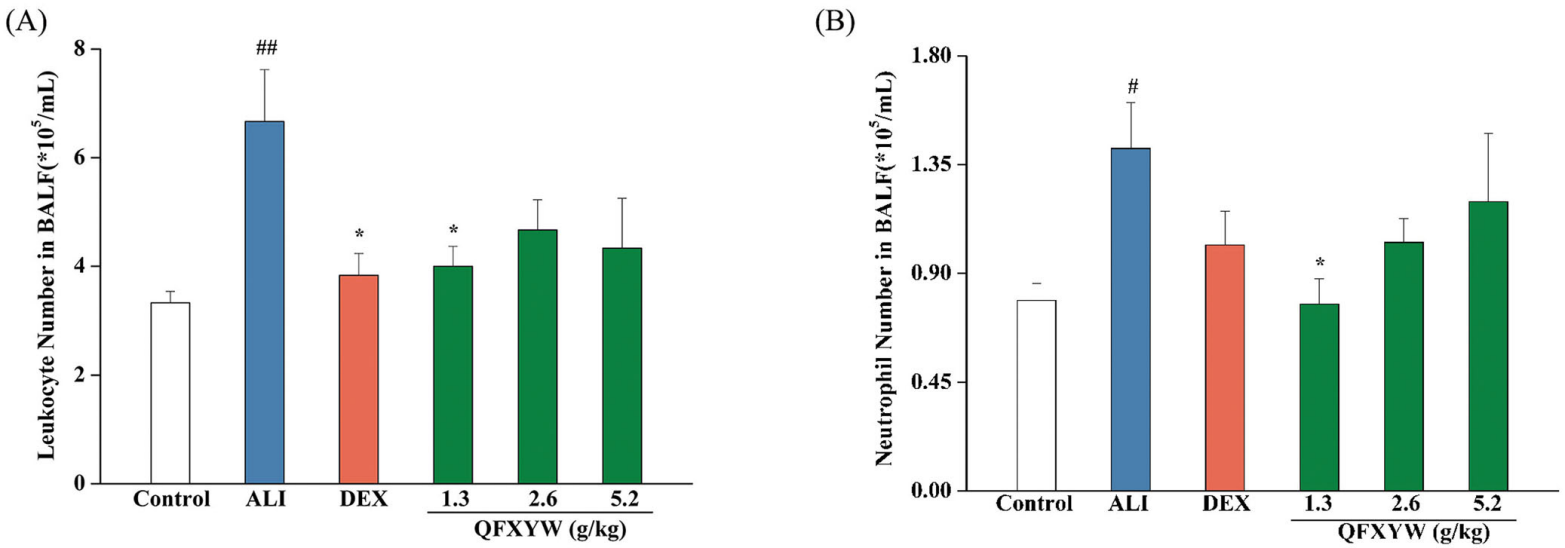
Effects of QFXYW on leukocytes (A) and neutrophils (B). (Compared with the control group, ^#^*p* < 0.01, ^##^*p* < 0.05; compared with the ALI group, *p < 0.05; *n* = 6). ALI: acute lung injury; QFXYW: Qingfei Xiaoyan Wan.

**Figure 2.**
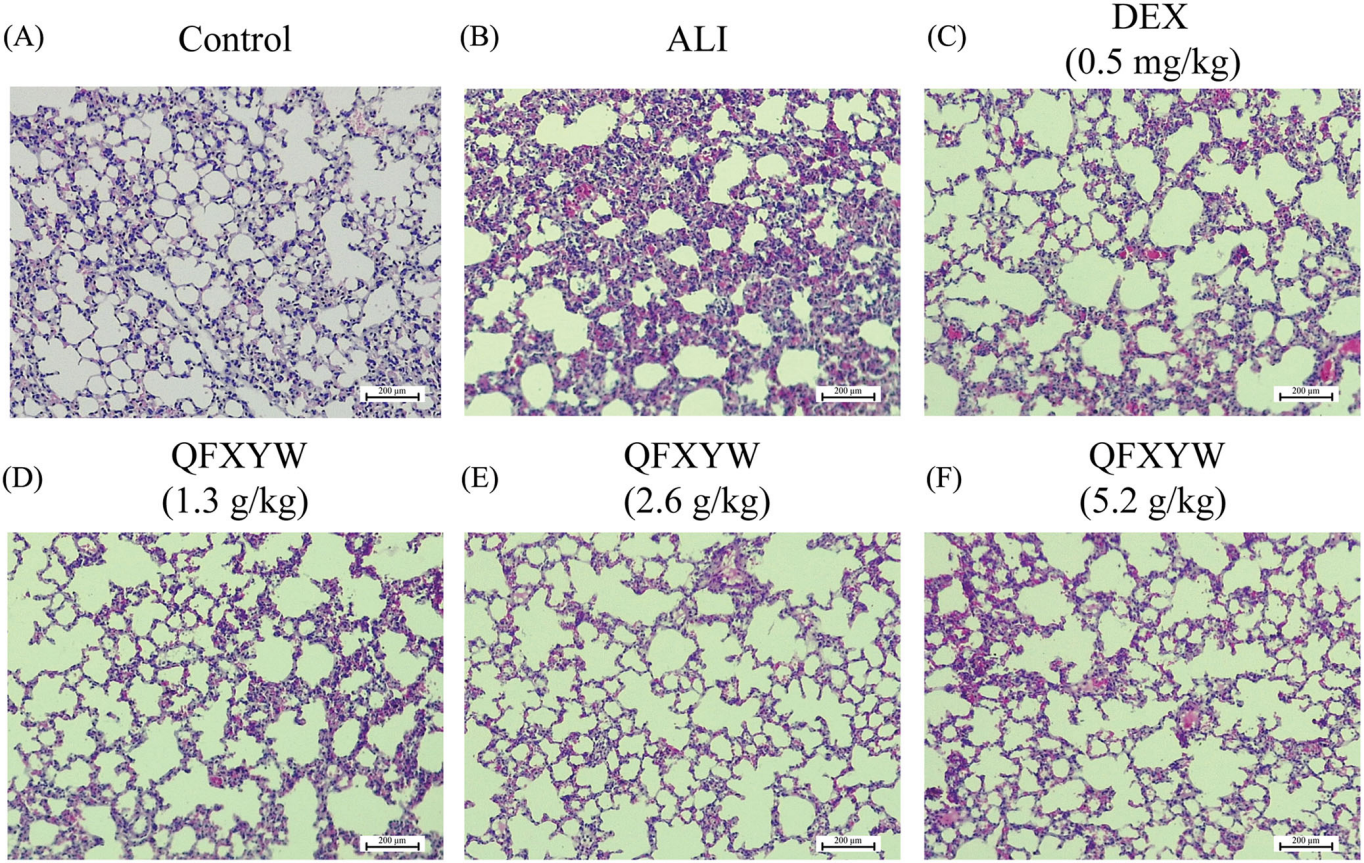
Histopathological changes of lung tissues of mice in each group (A) Control group; (B) ALI group; (C) DEX group; (D–F) QFXYW (1.3, 2.6, and 3.9 g/kg). ALI: acute lung injury; DEX: dexamethasone; QFXYW: Qingfei Xiaoyan Wan.

Among the types of leukocytes, neutrophils are the most abundant and their activation and recruitment are important markers of ALI. After LPS stimulation, the total number of leukocytes in the BALF of the ALI group increased significantly, compared with the control group (*p* < 0.01), while in the ALI + DEX and ALI + QFXYW groups, there was a reduction in the total number of leukocytes in the BALF (Figure 1A). Similarly, compared with the control group, LPS stimulation significantly increased the total number of neutrophils in the BALF (*p* < 0.05). After administration of QFXYW, the number of neutrophils in the BALF was reduced to varying degrees, suggesting that QFXYW could effectively suppress the inflammatory response of lung tissue by reducing the infiltration of inflammatory cells (Figure 1). Additionally, to further investigate the therapeutic effects of QFXYW, histopathological changes in the lung tissue were observed after staining with H&E. Without LPS stimulation, the alveolar structures in the lung tissues were clear, with normal spacing and tubular wall structure (Figure 2). In contrast, numerous histological changes, such as inflammatory cell infiltration, alveolar septal thickening, partial alveolar wall collapse, and exudation of red blood cells and protein fluid, were observed in the lung tissues after LPS stimulation. As per our initial hypothesis, we observed that similar to DEX, QFXYW could improve the inflammatory microenvironment in lung tissue to varying degrees.

### Transcriptomics analysis results of QFXYW-treated LPS-induced ALI in mice

**Figure 3.**
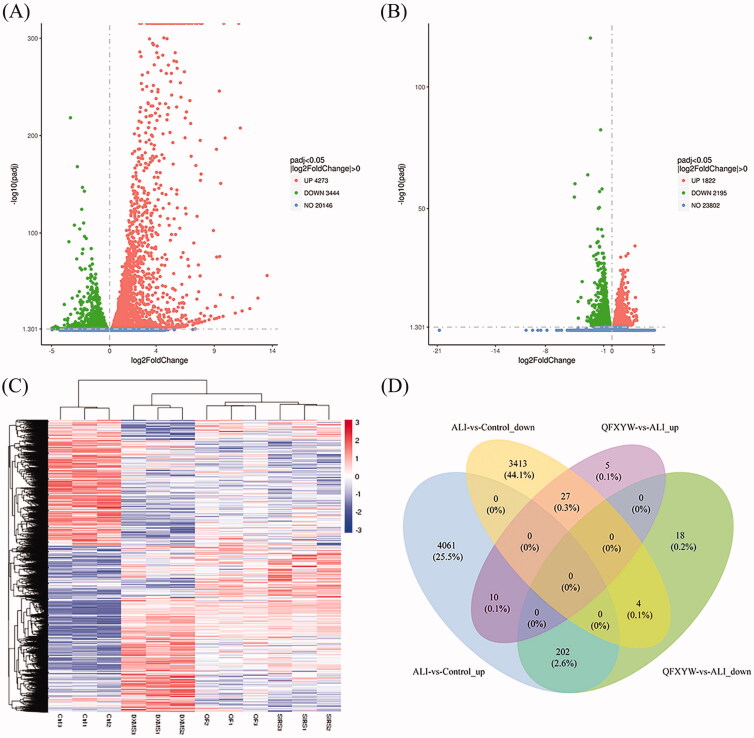
Transcriptomics analysis of QFXYW treated ALI. (A) Volcano map of the control group; (B) Volcano map of the ALI group; (C) Heatmap of the control, DEX, QFXYW, and ALI groups. (D) Venn diagram of differentially expressed genes. ALI: acute lung injury; DEX: dexamethasone; QFXYW: Qingfei Xiaoyan Wan.

In this study, transcriptomics analyses were performed to further explore the protective effect of QFXYW on ALI. The DEGs were screened based on a cut-off value of *p* < 0.05 (Figure 3A,B). Compared with the control group, there were 7717 DEGs in the ALI group; among these, 4723 were up-regulated genes and 3444 were down-regulated genes. A total of 266 DEGs were identified in the QFXYW vs. ALI group, 42 of which were up-regulated genes and 224 were down-regulated. Hierarchical cluster analysis was performed to compare the DEGs in each group. After QFXYW administration, partial gene expression disorders induced by LPS reversal were observed, indicating that QFXYW has a certain effect on LPS-induced transcription of lung tissue (Figure 3C). In order to clarify the distribution of DEGs in the treatment of ALI with QFXYW, the Venny online software was used to analyse the intersection of the upregulated and downregulated genes in the QFXYW vs. ALI and ALI vs. control groups. The results suggested that LPS could upregulate the expression of 4273 DEGs, while QFXYW could reverse the upregulation of 202 DEGs between the upregulated genes in the ALI vs. control group and downregulated genes in the QFXYW vs. ALI group. By comparing the downregulated and upregulated genes in the ALI vs. control and QFXYW vs. ALI groups, respectively, we also observed that the expression of 3414 DEGs was down-regulated after the ALI treatment, while the expression of 27 DEGs could be reversed after QFXYW administration.

### Network construction and analysis of DEGs

**Figure 4.**
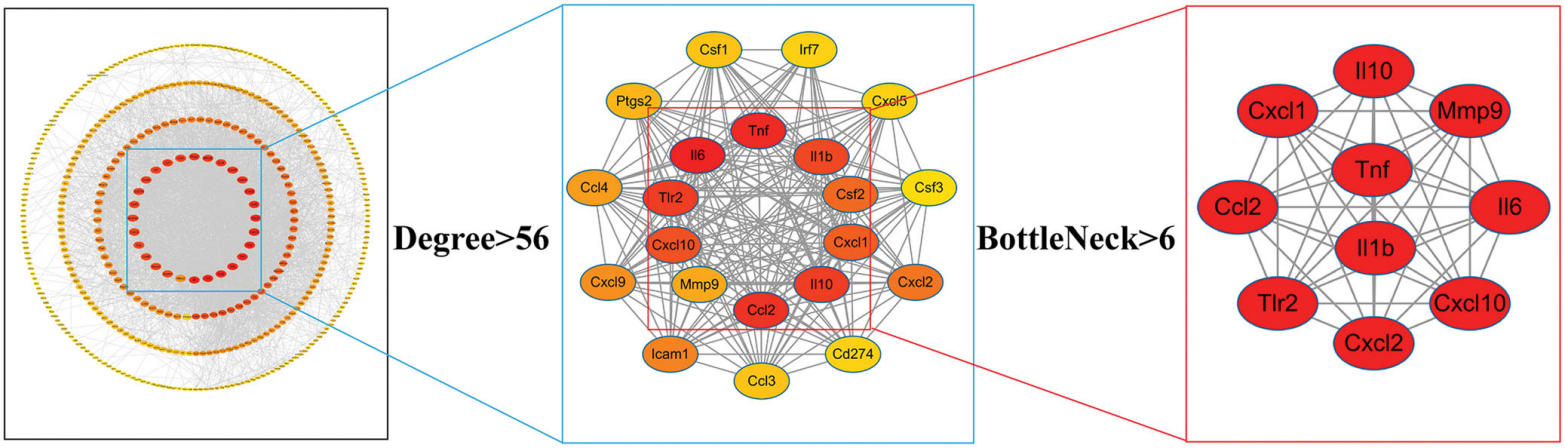
The process used for the screening of hub genes.

To include more DEGs related to the pathogenesis of ALI, we first imported the top 500 DEGs in the ALI vs. control group and 266 DEGs in the QFXYW vs. ALI group into the STRING database to construct a PPI network, which are 284 nodes and 3014 interaction edges in the PPI network of the ALI vs. control groups, 195 nodes and 698 interaction edges in the PPI network of the QFXYW vs. ALI groups. The two aforementioned PPI networks were combined to obtain the Merged network with 427 nodes and 3491 interaction edges. The following topological characteristic parameters of nodes in the network were subsequently calculated via CytoHubba: Degree of node connection (Degree), Edge Percolated Component, Maximum Neighbourhood Component, Eccentricity, Density of Maximum Neighbourhood Component, Maximal Clique Centrality, BottleNeck (BN), Closeness, Radiality, Betweenness, and Stress (Zhu and Hou 2020). Degree refers to the number of directly adjacent nodes; the number of directly connected nodes is directly proportional to the influence it exerts, which is a simple and intuitive way to use this parameter to measure the importance of a node (Racz et al. 2020). BN refers to nodes with high intermediateness, similar to the main bridges on expressways (Xu et al. 2020). Thus, the nodes with high Degree and BN were screened for the analysis. These important nodes with numerous connections play a pivotal role in the whole network and are considered hub genes (Prasad et al. 2020). For the screening process of the hub genes (Figure 4), the nodes in the network with a Degree >7 times of the median (Degree > 56) were used as the key network with 22 nodes and 215 interaction edges. Next, after screening the nodes with BN greater than the median value (BN > 6), 10 hub genes were obtained, namely, IL-6, TNF-α, CCL2, TLR2, IL-10, IL-1β, CXCL10, CXCL1, CXCL2, and MMP-9, were obtained. Among them, IL-6 and TNF-α had the highest Degree, with values of 178 and 141, respectively, and were considered as the most critical genes. The topological parameters of the hub genes are listed in Table 2.

**Table 2.** Topological parameter of hub genes.

No.	Genes	Protein names	Target class	Degree
1	CXCL10	C-X-C motif chemokine ligand 10	Signalling	30
2	CXCL2	C-X-C motif chemokine ligand 2	Signalling	30
3	CXCL1	C-X-C motif chemokine ligand 1	Signalling	29
4	IL-6	Interleukin 6	None	24
5	CCL5	C-C motif chemokine ligand 5	Signalling	23
6	CCL4	C-C motif chemokine ligand 4	Signalling	23

### Functional enrichment analysis

**Figure 5.**
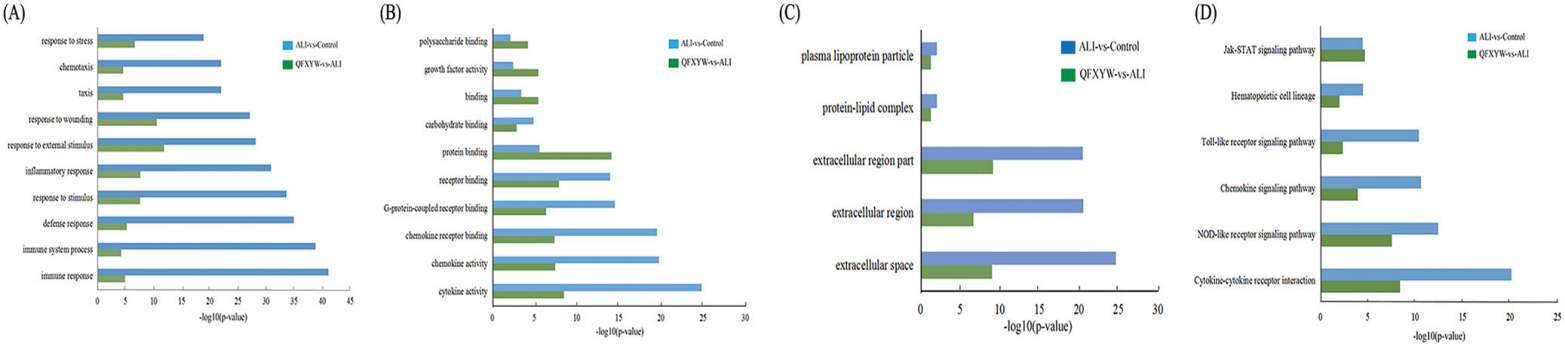
Functional enrichment analysis of DEGs between the control vs. ALI and QFXYW vs. ALI groups. (A) Biological process (BP) of DEGs in the control vs. ALI group and QFXYW vs. ALI group; (B) Cellular component (CC) of DEGs in the control vs. ALI group and QFXYW vs. ALI group; (C) Molecular function (MF) of DEGs in the control vs. ALI group and QFXYW vs. ALI group; (D) KEGG of DEGs in the control vs. ALI group and QFXYW vs. ALI group. ALI, acute lung injury; DEG: differentially expressed genes; KEGG: Kyoto Encyclopaedia of Genes and Genomes; QFXYW: Qingfei Xiaoyan Wan.

To further explore the GO terms involved in the treatment of ALI, the GO enrichment analysis was conducted with the top 266 DEGs and 500 DEGs in the QFXYW vs. ALI and ALI vs. control groups, respectively. Then, venny 2.1.0 (https://bioinfogp.cnb.csic.es) was used to overlap the common GO terms (Figure 5A–C), and it includes three parts: biological process (BP), molecular function (MF), and cellular component (CC). The BP analysis results showed that ALI vs. control and QFXYW vs. ALI significantly enriched 218 and 224 GO terms, respectively, among which 106 GO terms overlapped. Some of the top 10 common GO terms were immune response, immune system process, defense response, inflammatory response, response to stimulus, chemotaxis, etc. The MF analysis showed that ALI vs. control and QFXYW vs. ALI significantly enriched 38 and 25 GO terms, respectively, of which 13 GO terms overlapped. The top 10 overlapping GO terms involved cytokine activity, chemokine activity, chemokine receptor binding, G-protein-coupled receptor binding, growth factor activity, and protein binding. The CC analysis results showed that ALI vs. control and QFXYW vs. ALI significantly enriched 8 and 7 GO terms, respectively, of which 5 GO terms overlapped, which were extracellular space, extracellular region, protein-lipid complex, and plasma lipoprotein particle. Additionally, we performed the KEGG pathway analysis with the top 500 DEGs in the ALI vs. control and the top 266 DEGs in the QFXYW vs. ALI groups. ALI vs. control and QFXYW vs. ALI were significantly enriched in 12 and 13 pathways, respectively (Figure 5D); there were 6 common pathways, which were as follows: cytokine-cytokine receptor interaction, NOD-like receptor signalling pathway, chemokine signalling pathway, toll-like receptor (TLR) signalling pathway, haematopoietic cell lineage, and JAK-STAT signalling pathway. Among these enriched signalling pathways, the interaction between cytokine and cytokine receptor was the most significantly enriched pathway, and was consistent with the GO terms, among the ALI vs. control and QFXYW vs. ALI groups, which may play an important role in the treatment of ALI by QFXYW.

### RT-PCR validation of hub genes

**Figure 6.**
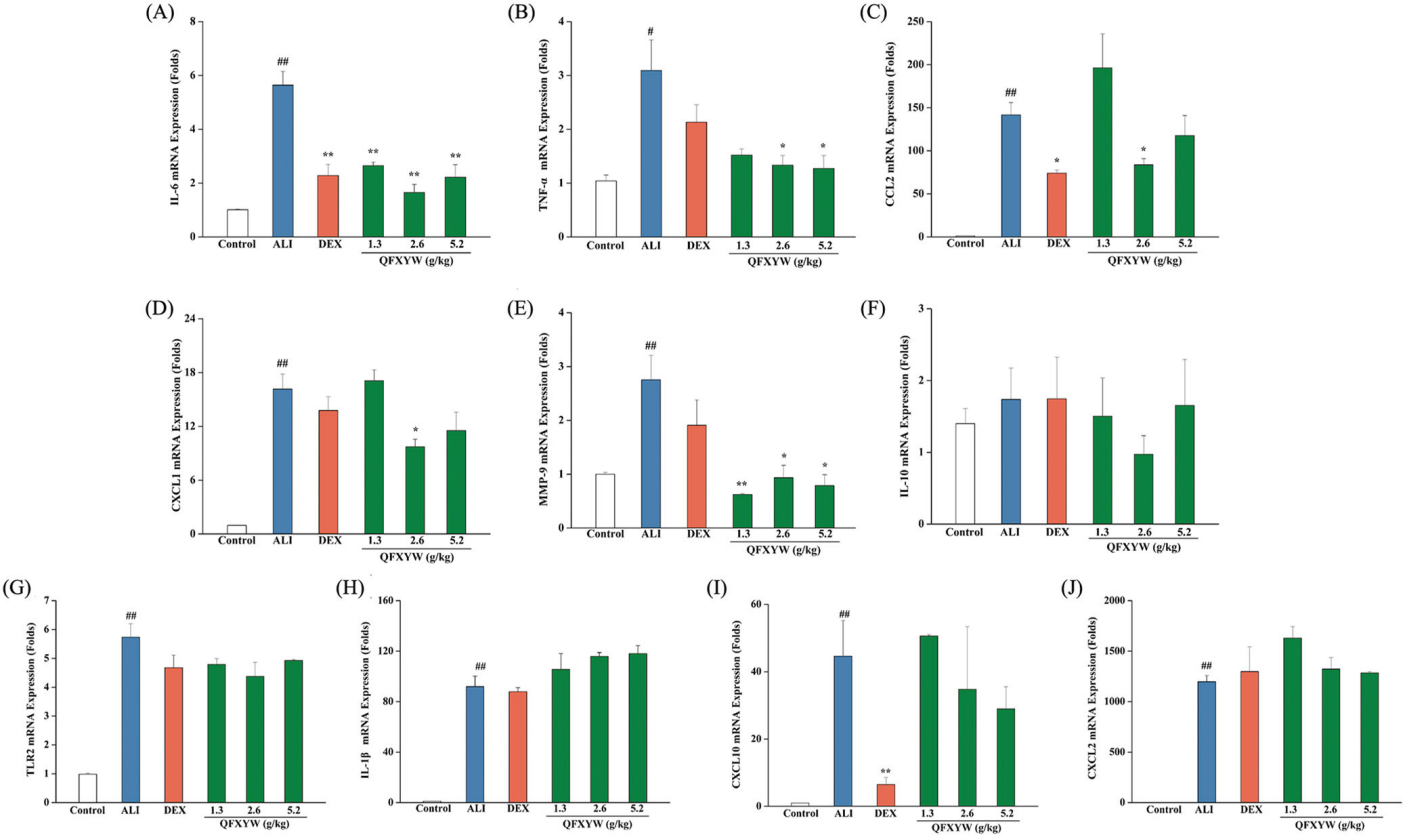
Effects of the QFXYW on the mRNA expression in LPS induced ALI (A) IL-6; (B) TNF-α; (C) CCL2; (D) CXCL1; (E) MMP-9; (F) IL-10; (G) TLR2; (H) IL-1β; and (I) CXCL10 (Compared with the control group, ^#^*p* < 0.01, ^##^*p* < 0.05; compared with the ALI group, *p < 0.05; *n* = 3) LPS, lipopolysaccharide; ALI: acute lung injury; QFXYW: Qingfei Xiaoyan Wan.

To further investigate the therapeutic effect of QFXYW on ALI at the gene expression level, the mRNA expression levels of IL-6, TNF-α, CCL2, TLR2, IL-10, IL-1β, CXCL10, CXCL1, CXCL2, and MMP-9 were detected. The results showed that QFXYW could significantly inhibit the mRNA expression levels of IL-6, TNF-α, and CCL2 in ALI-induced lung tissues. RT-PCR results showed that QFXYW could significantly inhibit the mRNA expression of CXCL1 and MMP-9, and QFXYW (2.6 g/kg) had a better inhibitory effect on IL-6, TNF-α, CXCL1, and MMP-9 than DEX. Unfortunately, the results demonstrated that QFXYW did not significantly inhibit the mRNA expression of IL-1, TLR2, CXCL10, and CXCL2 or significantly promote the mRNA expression of IL-10 (Figure 6).

### Validation of hub proteins using cytokines analysis

**Figure 7.**
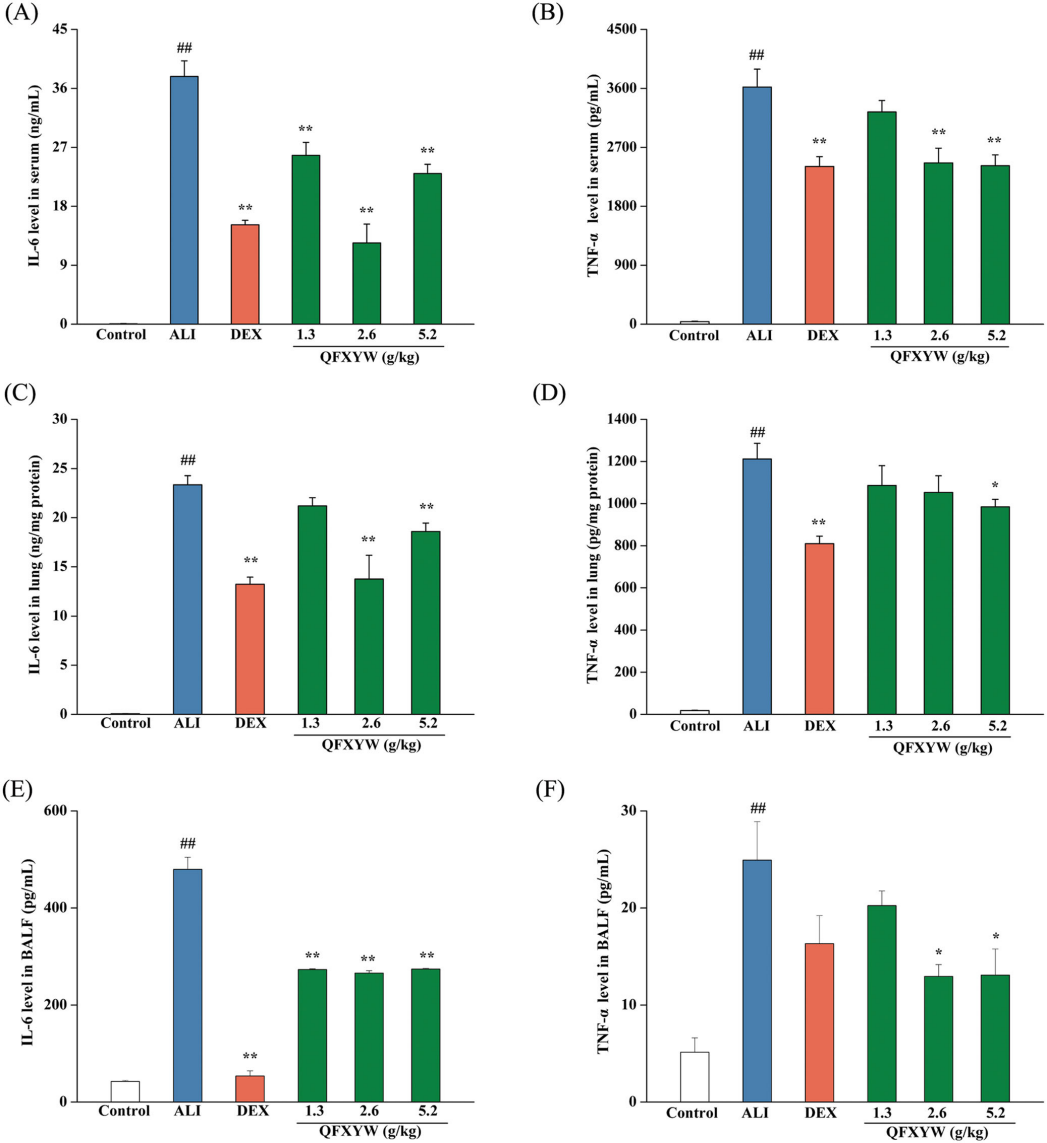
Effect of QFXYW on the protein expression of IL-6 and TNF-α. (A) IL-6 in the serum; (B) TNF-α in the serum; (C) IL-6 in the lung tissue; (D) TNF-α in the lung tissue; (E) IL-6 in the BALF; (F) TNF-α in the BALF. BALF: bronchoalveolar lavage fluid; QFXYW: Qingfei Xiaoyan Wan.

Pro-inflammatory cytokines, such as IL-6 and TNF-α, are considered to be the most promising biomarkers for ALI (Simons et al. 1996). To further explore the effects of QFXYW on IL-6 and TNF-α in ALI, the protein expression levels of IL-6 and TNF-α in the serum, BALF, and lung tissues were detected using ELISA. As shown in Figure 7, statistical analysis revealed that after LPS stimulation, IL-6 and TNF-α were secreted in large amounts (*p* < 0.01). Compared with the ALI group, different doses of QFXYW inhibited the increase of IL-6 and TNF-α in the serum, lung tissue, and BALF induced by LPS. DEX also significantly suppressed the increased concentration of IL-6 and TNF-α in the serum, while the medium-dose QFXYW group (2.6 g/kg) had a better inhibitory effect on IL-6 than the DEX group. In summary, it is suggested that QFXYW could effectively reduce the inflammatory response by inhibiting the protein expression of IL-6 and TNF-α in serum, BALF, and lung tissue.

### Verification of protein array expression

**Figure 8.**
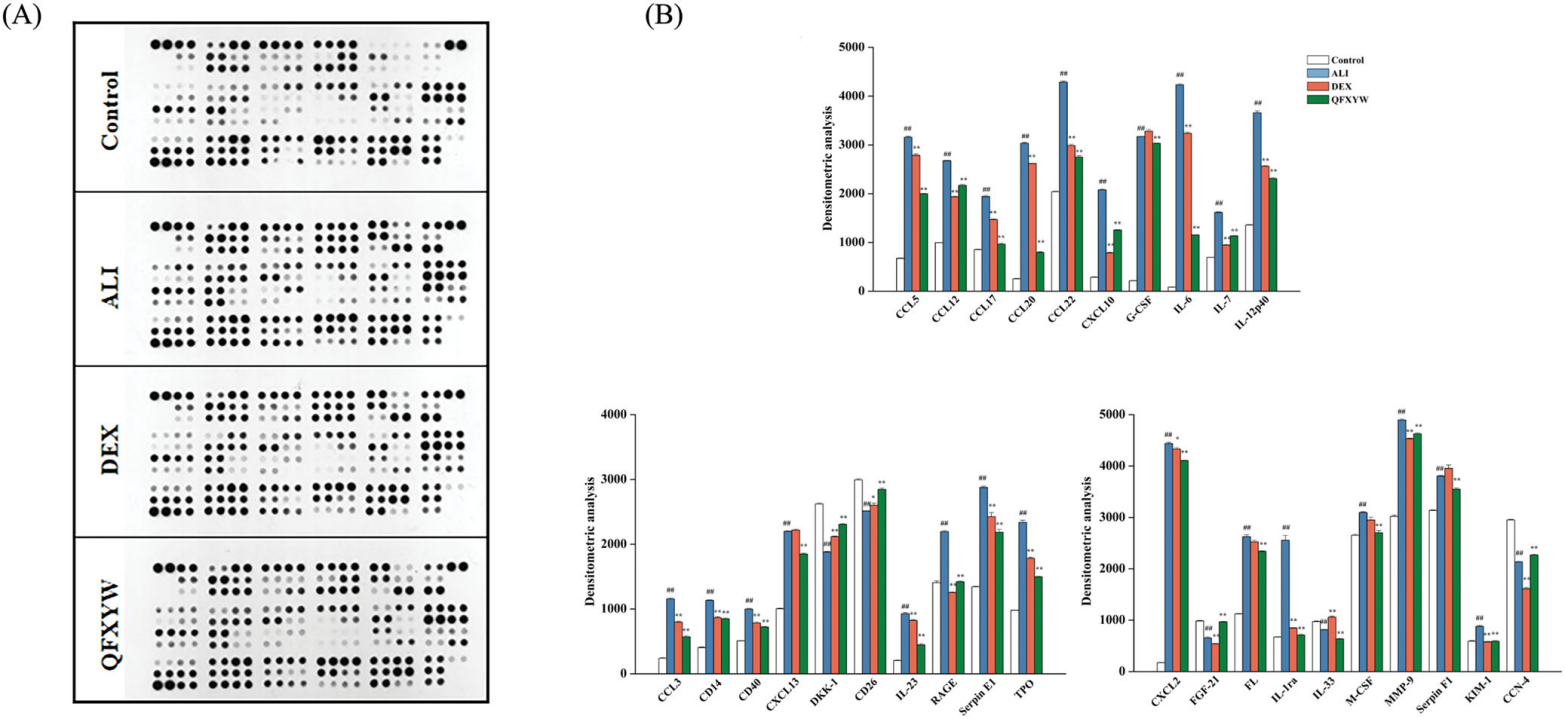
Effect of QFXYW on the production of multiple cytokines in the serum of the mice with ALI. (A) Cytokines and chemokines screened using cytokine protein assay; (B) Optical density detected by Image. ALI: acute lung injury; QFXYW: Qingfei Xiaoyan Wan.

**Figure 9.**
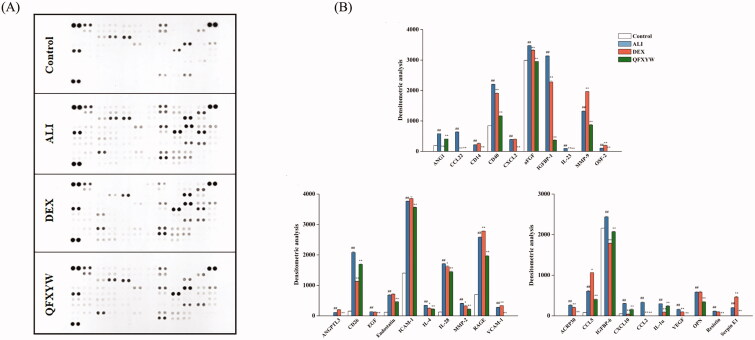
Effect of QFXYW on the production of multiple cytokines in the lung tissue of mice with ALI. (A) Cytokines and chemokines screened using cytokines protein assay; (B) Optical density detected by Image. ALI: acute lung injury; QFXYW: Qingfei Xiaoyan Wan.

Functional enrichment analysis showed that the cytokine signalling pathway is one of the important pathways for QFXYW to treat ALI. Therefore, a cytokine protein chip was used to analyse the effect of QFXYW on the expression of various cytokines in the serum with LPS-induced ALI. The top 30 cytokines in the serum were screened, and the significance was set at *p* < 0.05 for comparisons between the ALI vs. control and the QFXYW vs. ALI groups (Figure 8). The results exhibited that LPS significantly upregulated the protein expression of CCL5, CCL12, CCL17, CCL20, CCL22, CXCL10, G-CSF, IL-6, IL-7, IL-12p40, CCL3, CD14, CD40, CXCL13, IL-23, RAGE, Serpin E1, TPO, CXCL2, FL, IL-1ra, M-CSF, MMP-9, Serpin F1, and KIM-1, while it downregulated the protein expressions of DKK-1, CD26, FGF-21, IL-33, and CCN4. Administration of QFXYW (2.6 g/kg) significantly inhibited the overexpression protein of CCL5, CCL12, CCL17, CCL20, CCL22, CXCL10, G-CSF, IL-6, IL-7, IL-12p40, CCL3, CD14, CD40, CXCL13, IL-23, RAGE, Serpin E1, TPO, CXCL2, FL, IL-1ra, M-CSF, MMP-9, Serpin F1, and up-regulated DKK-1, CD26, FGF-21, and CCN4. Additionally, the effect of QFXYW on the expression of cytokine proteins in ALI lung tissues was also detected. The protein expression in lung tissues from each group is shown in Figure 9A, and the results are consistent with the expression in serum. Compared with the control group, LPS also significantly increased the expression of various inflammatory cytokines in the lung tissue, and the DEX and QFXYW groups significantly inhibited the over-expression of the various cytokine proteins. LPS significantly increased the expression of ANG1, CCL22, CD14, CD40, CXCL2, AFGF, IGFBP-1, IL-23, MMP-9, OSF-2, ANGPTL3, CD26, EGF, Endostatin, ICAM-1, IL-4, IL-28, MMP-2, RAGE, VCAM-1, ACRP30, CCL5, IGFBP-6, CXCL10, CCL2, IL-1α, VEGF, OPN, resistin, and serpin E1, while the QFXYW (2.6 g/kg) reversed the overexpression of these proteins (Figure 9B).

## Discussion

ALI is a systemic inflammatory reactive disease accompanied by alveolar-capillary membrane injury, enhanced vascular permeability, increased neutrophils count, and pulmonary edema. Owing to the high morbidity and mortality rates, ALI places a heavy burden on society and individuals (Eves et al. 2013; Yazicioglu et al. 2020). Previous studies have shown that several anti-inflammatory drugs (glucocorticoids, ketoconazole, and so on) have better efficacy in ALI; however, these drugs have not been converted into specific therapeutic drugs on account of the varied and substantially adverse effects (Pearce et al. 2006). Considering its strong activity, novel structure, multi-target action, low toxicity, and safety, TCM has become an important direction for exploring novel drugs for complex diseases (Hui et al. 2020; Zhang et al. 2021). QFXYW is derived from the classic formula “Maxing Shigan Decoction,” approved by the China Food and Drug Administration (NO. Z12020757), and has good anti-inflammatory, anti-bacterial, anti-viral, anti-asthma, and other effects (Cheng et al. 2012; Zhao et al. 2013). However, owing to the complexity and diversity of the ingredients of the TCM formula, it has been relatively difficult to investigate the efficacy of TCM for complex diseases. In recent years, the development of peripheral subjects, such as system pharmacology (Cao et al. 2021), transcriptomics, and proteomics (Arif et al. 2020), has enabled researchers to further investigate the pathological changes in the treatment of complex diseases using the TCM formula. In this study, we applied transcriptomics integrated with system pharmacology to further investigate the potential mechanism of QFXYW in the treatment of ALI.

Given that LPS-induced ALI is a well-recognized and reproducible experimental model, it covers the significant symptoms of ALI, such as alveolar epithelial cell rupture, endothelial cell function injury, inflammatory infiltration of cells, and local inflammatory response. Thus, we selected LPS-induced ALI as the model for the subsequent investigation. After successfully developing an LPS-induced ALI model, different doses of QFXYW (1.3, 2.6, and 5.2 g/kg) were administered to observe the changes in the leukocyte and neutrophil counts and pathological characteristics. We observed that QFXYW could reverse the damage caused by LPS to various degrees, indicating that QFXYW has a protective effect on ALI.

Transcriptomics mainly focuses on the discipline of transcription and regulation of all genes in the biological process, establishing a connection between the genetic information of the genome and the biological function of the proteome (Ouattara et al. 2020). Next-generation sequencing (RNA-seq) was applied for the transcriptional analysis of the lung tissue, enabling high-throughput and detailed characterisation of gene expression profiles at the tissue level, which has the potential to identify characteristic gene expression in complex diseases such as ALI and asthma. The transcriptomic results of lung tissues demonstrated that there was a total of 7717 DEGs and 266 DEGs in the control vs. ALI and ALI vs. QFXYW groups, respectively, suggesting that QFXYW exerts a therapeutic effect through multiple genes, instead of a single gene. To further elucidate the mechanism of QFXYW in the treatment of ALI, a PPI network of DEGs was constructed. The Degree and BN, important parameters of the network topology, were applied as the criteria for screening the hub genes, and 10 hub genes were identified, namely, IL-6, TNF-α, CCL2, TLR2, IL-10, IL-1β, CXCL10, CXCL1, CXCL2, and MMP-9, were identified. Additionally, RT-PCR was performed to verify the gene expression level of the hub genes, and the results showed that compared with the ALI group, QFXYW could significantly inhibit the gene expression levels of IL-6, TNF-α, CCL2, CXCL1, and MMP-9, while the inhibitory effects on the gene expression of TRL2, IL-1β, CXCL10, and CXCL2 was not obvious. Among these hub genes, TLR2 is an inflammation-related receptor that is involved in inflammation-induced diseases, especially that induced by LPS, which serves as a transduction and transmembrane molecule (Wang et al. 2016). Key inflammatory cytokines (IL-6 and TNF-α) have been reported to play a key role in the early stage of ALI (Simons et al. 1996). Cytokines not only participate in the recruitment of neutrophils into the lung and enhance the neutrophil response to activators, but also cause severe inflammatory cascade injury (Sun et al. 2020). Additionally, CCL2 is an important pro-inflammatory cytokine that is secreted by a variety of cells during inflammation in the body and has a specific chemotactic activation effect on monocytes/macrophages (Peng et al. 2020). Moreover, in the process of ALI, promoting the release of chemokines is also an important link in the inflammatory response of ALI. CXCL1 and CXCL2 are considered the most important chemokines for neutrophil recruitment. MMP-9 is involved in neutrophil migration through the middle membrane; although it may not be involved via the endothelium (Xu et al. 2019), which may also promote the accumulation of neutrophils by activating or inactivating pro-inflammatory cytokines and chemokines (Jan et al. 2019).

Functional enrichment analyses have demonstrated that the potential mechanism of QFXYW in the treatment of ALI mainly involves the cytokine-cytokine signalling pathway, along with multiple signalling pathways, such as the TLR signalling pathway, and JAK-STAT signalling pathway. Considering the aforementioned predicted results, we performed the cytokine protein chip assay to verify the protein expression of multiple cytokines in the serum and lung tissues. The protein array technology enabled us to perform multiple western blotting for the semi-quantitative detection of the expression of 111 protein (Dai et al. 2018). The results showed that QFXYW (2.6 g/kg) could inhibit the protein expression of CCL5, CCL12, CCL17, CCL20, CXCL10, IL-6, and so on, indicating that QFXYW reverses the injury of ALI via multiple targets and pathways. Additionally, TLRs have special functions in lung-related diseases; for example, the activation of TLRs leads to the secretion of key inflammatory cytokines (IL-6 and TNF-α) (Ge et al. 2020), which have been reported to play a key role in early-stage ALI. Previous studies have shown that the JAK-STAT signalling pathway is regulated by a variety of cytokines, exerting a critical role in lung injury and immune mediation (Zheng and Li 2018).

## Conclusions

We evaluated the therapeutic mechanism of QFXYW in the treatment of ALI by integrating transcriptomics analysis, system pharmacology, and *in vivo* experiments. We observed that QFXYW exerted a protective effect against ALI in mice through the involvement of multiple hub genes (IL-6, TNF-α, CCL2, CXCL1, and MMP-9) and key pathways (cytokine-cytokine signalling pathway, TLR signalling pathway, and JAK-STAT signalling pathway), which provides a scientific basis for the elucidation of the pathological mechanism of ALI. We believe that further research on this subject may help incorporate QFXYW into the treatment protocol for ALI, thus providing an alternative that is relatively safer compared to conventional medicines.
